# Yangjing capsule improves oligoasthenozoospermia by promoting nitric oxide production through PLCγ1/AKT/eNOS pathway

**DOI:** 10.3389/fphar.2023.1056091

**Published:** 2023-04-26

**Authors:** Weimin Deng, Dalin Sun, Bin Cai, Baofang Jin

**Affiliations:** Zhongda Hospital Southeast University, Nanjing, China

**Keywords:** oligoasthenozoospermia, nitric oxide, PLCγ1/AKT/eNOS, yangjing capsule, ornidazole

## Abstract

**Background:** Oligoasthenozoospermia is an important factor leading to male infertility. Yangjing capsule (YC), a traditional Chinese preparation, displays beneficial effects on male infertility. However, whether YC could improve oligoasthenozoospermia remains unclear.

**Methods:** In this study, we aimed to explore the effect of YC in the treatment of oligoasthenozoospermia. Male Sprague-Dawley (SD) rats were treated with 800 mg/kg ornidazole once daily for 30 days to induce *in vivo* oligoasthenozoospermia; primary Sertoli cells were treated with 400 μg/mL ornidazole for 24 h to induce *in vitro* oligoasthenozoospermia.

**Results:** We found that YC improved the testicle and epididymis weight, sperm concentration, sperm progressive motility, serum testosterone, fertility rate and testis morphology in ornidazole-exposed rats and enhanced cell survival in ornidazole-stimulated primary Sertoli cells. YC also inhibited the ornidazole-caused decrease in nitric oxide (NO) generation and the phosphorylation of phospholipase C γ1 (PLCγ1), AKT, and eNOS *in vivo* and *in vitro* in oligoasthenozoospermia. Furthermore, the knockdown of PLCγ1 blunted the beneficial effects of YC *in vitro*.

**Conclusion:** Collectively, our data suggested that YC protected against oligoasthenozoospermia by promoting NO levels through the PLCγ1/AKT/eNOS pathway.

## Introduction

Infertility refers to the inability of a non-contracepting couple in accessing pregnancy after 12 months of regular sexual intercourse (Maghsoumi-Norouzabad et al., 2021). It is a severe problem that affects an estimated 15% of couples of childbearing age worldwide, and males account for approximately 50% of infertile individuals (Lu et al., 2020). Oligoasthenozoospermia, which is characterized by low sperm concentration and motility, is considered one of the major causes of male infertility (Zou et al., 2019). However, there is no effective drug for the treatment of oligoasthenozoospermia in modern medicine to date (Zhao et al., 2018).

Nitric oxide (NO) is a reactive nitrogen species produced by three isoforms of nitric oxide synthase (NOS), namely, neuronal nitric oxide synthase (nNOS), endothelial nitric oxide synthase (eNOS), and inducible nitric oxide synthase (iNOS) (Pradhan et al., 2018). NO participates as a mediator in several biological and physiological processes, including male reproductive function. Previous evidence suggests ayurvedic botanical drug-induced NO production contributes to the improvement of sperm count, penile erection, and seminal fructose levels in rats (Thakur et al., 2011). Wang et al. monitored NO concentration changes during sperm capacitation *in vitro* and noticed that the abnormal semen group spent more time reaching an appropriate NO concentration relative to the control group (Wang et al., 2014). Adrenomedullin, a polypeptide, enhances sperm motility by improving nitric oxide levels in the spermatozoa (Chiu et al., 2010). AKT is a serine/threonine kinase that has been demonstrated to influence NO production by targeting eNOS signaling (Kumar and Mani, 2021). Urocortin 2, a cardioactive peptide, stimulates nitric oxide generation in ventricular myocytes via AKT-mediated phosphorylation of eNOS at serine 1,177 (Walther et al., 2014). The activation of the AKT/eNOS pathway induced by baicalin treatment promotes NO levels in rats that underwent myocardial ischemia-reperfusion (Bai et al., 2019). Phospholipase C γ1 (PLCγ1), which can bind to AKT, is a member of the PLC family that functions as a signal transducer by hydrolyzing membrane lipids to generate second messengers (Chen and Simons, 2021). Emerging evidence suggests that PLCγ1 plays a crucial role in the AKT/eNOS pathway-related NO production (Yu et al., 2017).

The Yangjing capsule (YC) is a traditional Chinese preparation that consists of 11 drugs, including Herba Epimedii Brevicornus, Semen Vaccariae Segetalis, Concha Ostreae (calcined), Radix Angelicae Sinensis, Radix Astragali Mongolici, Semen Litchi, Placenta Hominis, Rhizoma Polygonati Sibirici, Radix Rehmanniae Preparata, Semen Astragali Complanati, and Hirudo. Existing evidence suggests that YC exerts beneficial effects on male reproductive diseases. YC improves the function of testicular angiogenesis by activating the eNOS pathway (Jin et al., 2020). One month of YC treatment ameliorates spermatogenesis in mice exposed to cyclophosphamide (Zhao et al., 2015). Additionally, the stimulative effect of YC on testosterone synthesis was observed in Leydig cells (Gu et al., 2015). YC also significantly promoted sperm motility in patients with idiopathic asthenozoospermia when compared to the controls (Shen et al., 2010). Yangiing capsule plus low-dose tadalafil is demonstrated to be safe and effective for the treatment of functional anejaculation (Jin et al., 2012). However, whether YC could mitigate oligoasthenozoospermia remains unclear. In the current study, we established *in vivo* and *in vitro* models of oligoasthenozoospermia to explore the effects of YC on oligoasthenozoospermia.

## Materials and methods

### Preparation of YC

YC is composed of 11 Chinese medicines: *Epimedium sagittatum* (Siebold and Zucc.) Maxim. [Berberidaceae], *Gypsophila vaccaria* (L.). Sm. [Caryophyllaceae], Concha Ostreae (calcined), *Angelica sinensis* (Oliv.) Diels [Apiaceae], *Astragalus mongholicus* Bunge [Fabaceae], *Litchi chinensis* Sonn. [Sapindaceae], Placenta Hominis (sheep placenta), *Vitex negundo* L. [Lamiaceae], *Rehmannia glutinosa* (Gaertn.) DC. [Orobanchaceae], *Phyllolobium chinense* Fisch. [Fabaceae], Hirudo. The ratios of these medicines are 13.3: 13.3: 13.3: 10: 10: 6.7: 6.7: 6.7: 6.7: 6.7: 6.7. A total of 100 g YC crude drugs were immersed in 10 times (v/w) of water, heated, and boiled for 60 min. The filtrate was collected, concentrated with rotary evaporation at 60 °C until a final volume of 100 mL, and then lyophilized with a freeze dryer to get the extract (16.3 g). YC extract was diluted into 0.1 or 0.2 g/kg (expressed as gram extract per kilogram body weight) using normal saline for *in vivo* experiments and diluted into 1.6, 16, and 160 μg extract/mL using PBS solution for *in vitro* studies. The main components of YC, including ferulic acid (HY-N0820, MCE), catalpol (HY-N0820, MCE), complanatuside (HY-N0820, MCE), arctiin (HY-N0820, MCE), hyperoside (HY-N0820, MCE), and calycosin-7-O-β-D-glucoside (HY-N0820, MCE), were measured by HPLC-PDA (Waters Corporation, United States).

### Animals and treatment

Male Sprague-Dawley (SD) rats were purchased from Qinglongshan Laboratory Animal Company and housed in specific pathogen-free conditions with a 12-h light/12-h dark cycle at 22 °C. Protocols for animal experiments were approved by the Institution Animal Care and Use Committee of Southeast University (approval no. 20180309010). The animals were acclimated for 1 week prior to the *in vivo* experiments.

After adaption, the rats were randomly divided into five groups (*n* = 12 per group) as follows: (1) normal control rats (Saline), (2) ornidazole-induced oligoasthenozoospermic rats (Ornidazole); (3) ornidazole-induced oligoasthenozoospermic rats treated with 0.1 g/kg YC; (4) ornidazole-induced oligoasthenozoospermic rats treated with 0.2 g/kg YC and (5) ornidazole-induced oligoasthenozoospermic rats treated with levocarnitine (0.1 g/kg Lev). The rats in the Ornidazole, YC, and Lev groups were administered 800 mg/kg ornidazole once daily for 30 days to induce oligoasthenozoospermia. YC and levocarnitine were given once a day from day 1 to day 50. On day 37, partial rats (*n* = 6) were euthanized by decapitation. Blood was extracted by intracardiac puncture from rats, and serum samples were obtained by centrifugation for 10 min at 3,000 rpm and 4°C after blood coagulation. Bilateral testes and epididymides were also harvested for further use. The rest of the rats (*n* = 6) were subjected to the fertility assay until the end of the drug administration course. The saline group was treated with a 1% carboxymethylcellulose sodium (CMC-Na) solution and normal saline. All drugs were administered via oral gavage. The specific modeling and dosing designs are indicated in Table 1. Levocarnitine is the preferred drug for the treatment of male infertility syndrome; it was therefore used as a positive control in this study (Wang et al., 2021).

**TABLE 1:** Modeling and dosing design.

Group (*n* = 12)	Modeling (D1-30)	Dosing (D1-50)	D37
Saline	1% CMC-Na, i.g., once daily	normal saline, i.g., once daily	sampling (*n* = 6); start of fertility test (*n* = 6)
Ornidazole	800 mg/kg ornidazole, i.g., once daily	normal saline, i.g., once daily	
YC low dose	800 mg/kg ornidazole, i.g., once daily	0.1 g/kg, i.g., once daily	
YC high dose	800 mg/kg ornidazole, i.g. once daily	0.2 g/kg, i.g., once daily	
Levocarnitine	800 mg/kg ornidazole, i.g. once daily	0.1 g/kg, i.g., once daily	

Note: i.g., intragastrically. Modeling was performed at 8 a.m., and dosing was performed at 2 p.m.

### Isolation of sertoli cells

Sertoli cells were isolated from the testes of male neonatal SD rats. Briefly, the testis tissues were harvested, digested with 2 mg/mL type IV collagenase for 15 min, and incubated in 0.25% trypsin and 1 mM ethylene diamine tetra-acetic acid (EDTA) for 10 min at 37°C. The digestion was stopped with serum, then the cells were washed twice with 10% FBS fetal bovine serum (FBS) Dulbecco’s modified Eagle medium (DMEM) medium containing 1% streptomycin/penicillin. Next, the cell suspension was filtered through a 40-µm sterile nylon mesh, resuspended in 10% FBS DMEM medium containing 1% streptomycin/penicillin, and cultured in an incubator at 37°C for 2–3 days to allow for cell attachment. The debris and unattached Sertoli cells were washed away. Thereafter, cells were kept in culture for further use.

### Treatment of sertoli cells

Primary Sertoli cells were pretreated with YC (1.6, 16, 160 μg extract/mL) for 24 h and then cultured with 400 μg/mL ornidazole for another 24 h. Afterwards, the cells were collected for further analysis.

To explore the underlying mechanism, siRNA (5′-AAA​CCA​AGG​CUG​AGA​AGU​A-3′) directed specifically against PLCγ1 was transfected into Sertoli cells using Lipofectamine 2000 (Invitrogen) following the manufacturer’s protocol. At 48 h after transfection, cells were treated with 160 μg YC extract/mL for 24 h, followed by 24 h of 400 μg/mL ornidazole stimulation. Thereafter, cells were collected for further analysis.

### Sperm count and motility analysis

Epididymal sperm count and motility assay were performed as described before (Zhao et al., 2015). The epididymis was minced in physiological saline (1 mL; prewarmed at 37°C), and incubated for 15 min at 37°C to allow the sperm to swim out of the epididymal tubules. The sperm concentration and motility were determined using a hemocytometer. The sperm progressive motility was expressed as the ratio between the number of grade (A + B) sperms and the total number of sperms.

### Serum testosterone assay

The levels of testosterone in the serum of rats were measured by using a testosterone ELISA kit (Boster; EK7014) according to the manufacturer’s instructions. The data were expressed as ng of testosterone per mL of serum.

### Fertility study

To assess male fertility, two female rats were introduced per male. The females were checked daily for the presence of vaginal plugs, an indication of successful mating. After 2 weeks, the males were removed, and the females were maintained until spontaneous delivery. The number of litters was counted, and the fertility rate was calculated using the following equation: fertility rate = the number of females that gave birth/number of copulated females × 100%.

### Testicular histology

Testicular histopathology was assessed by hematoxylin and eosin (H&E) staining as previously described (Ma et al., 2021). Briefly, following fixation in Bouin’s solution for 6 h, testes were transferred to ethanol and xylene, embedded in paraffin, and sectioned at a thickness of 5 μm. Afterwards, the slices were stained with an H&E staining solution. Images of the sections were taken by light microscopy.

### Immunofluorescent staining

The immunofluorescent staining was performed as described elsewhere (Zheng et al., 2021). Cells or tissues were fixed in 4% paraformaldehyde, permeabilized with 0.5% Triton X-100, and blocked by incubation in 5% bovine serum albumin (BSA) for 1 h at 37°C. Then, the samples were incubated with primary antibodies against p-PLCγ1 (Tyr783; Affinity; AF3210; 1:100) or SOX9 (Abcam; ab185966; 5 μg/mL) overnight at 4°C. Next, a secondary antibody conjugated to Alexa Fluor^®^488 (Abcam; ab150077; 1:500) or Alexa Fluor^®^594 (Abcam; ab150080; 1:500) was added and incubated for 1 h at room temperature. After being rinsed with PBS, the samples were stained with DAPI, observed under an Olympus BX53 microscope, and analyzed using ImageJ.

### Determination of NO levels

The production of nitric oxide (NO) was measured by a commercial kit (Nanjing Jiancheng Bioengineering Institute; A013-2-1) as per the manufacturer’s instructions.

### Western blot assay

The western blot analysis was performed using a previously reported protocol (Guo et al., 2020). Cells or tissues were disrupted in a RIPA buffer containing protease and phosphatase inhibitors. After protein quantification with a bicinchoninic acid (BCA) kit, proteins were separated using 12% sodium dodecyl sulfate-polyacrylamide gel electrophoresis (SDS-PAGE). Gels were electroblotted onto polyvinylidene fluoride (PVDF) membranes. Non-specific binding was blocked using 5% BSA for 1 h at room temperature. Then the membranes were incubated with primary antibodies at 4°C overnight and probed with horseradish peroxidase-conjugated secondary antibodies for 90 min at room temperature. Enhanced chemiluminescence was used to amplify the protein signals. The density of immunoreactive proteins was assessed by using the ImageJ software. The following primary antibodies were used: PLCγ1 (Cell Signaling Technology; 5,690; 1:1000), p-PLCγ1 (Tyr783; Cell Signaling Technology; 14,008; 1:1000), eNOS (Cell Signaling Technology; 32,027; 1:1000), p-eNOS (Ser1179; ThermoFisher Scientific; PA5-105824; 1:1000), AKT (Cell Signaling Technology; 9,272; 1:1000), p-AKT (Ser473; Cell Signaling Technology; 4,060; 1:2000), GAPDH (Affinity; AF7021; 1:5,000).

### MTT

Methyl thiazolyl tetrazolium (MTT) was used to analyze the viability of cells (Zhang et al., 2019). After pretreatment with different concentrations of ornidazole or YC extract for 24 h, 10 μL of MTT solution (5 mg/mL) was added to each well and incubated for 4 h. Then, 150 μL of dimethyl sulfoxide (DMSO) solution was used to dissolve the formazan crystals. The absorbance at 570 nm was recorded by a microplate reader.

### Statistical analysis

All data are given as the mean ± standard deviation (SD). Differences between multiple groups were evaluated by one-way or two-way analysis of variance (ANOVA), followed by Dunnett’s post-test. Two-group analysis was compared by the unpaired t-test. The minimum level of significance was a *p*-value of <0.05.

## Results

### Chemical profiling of YC

**FIGURE 1:**
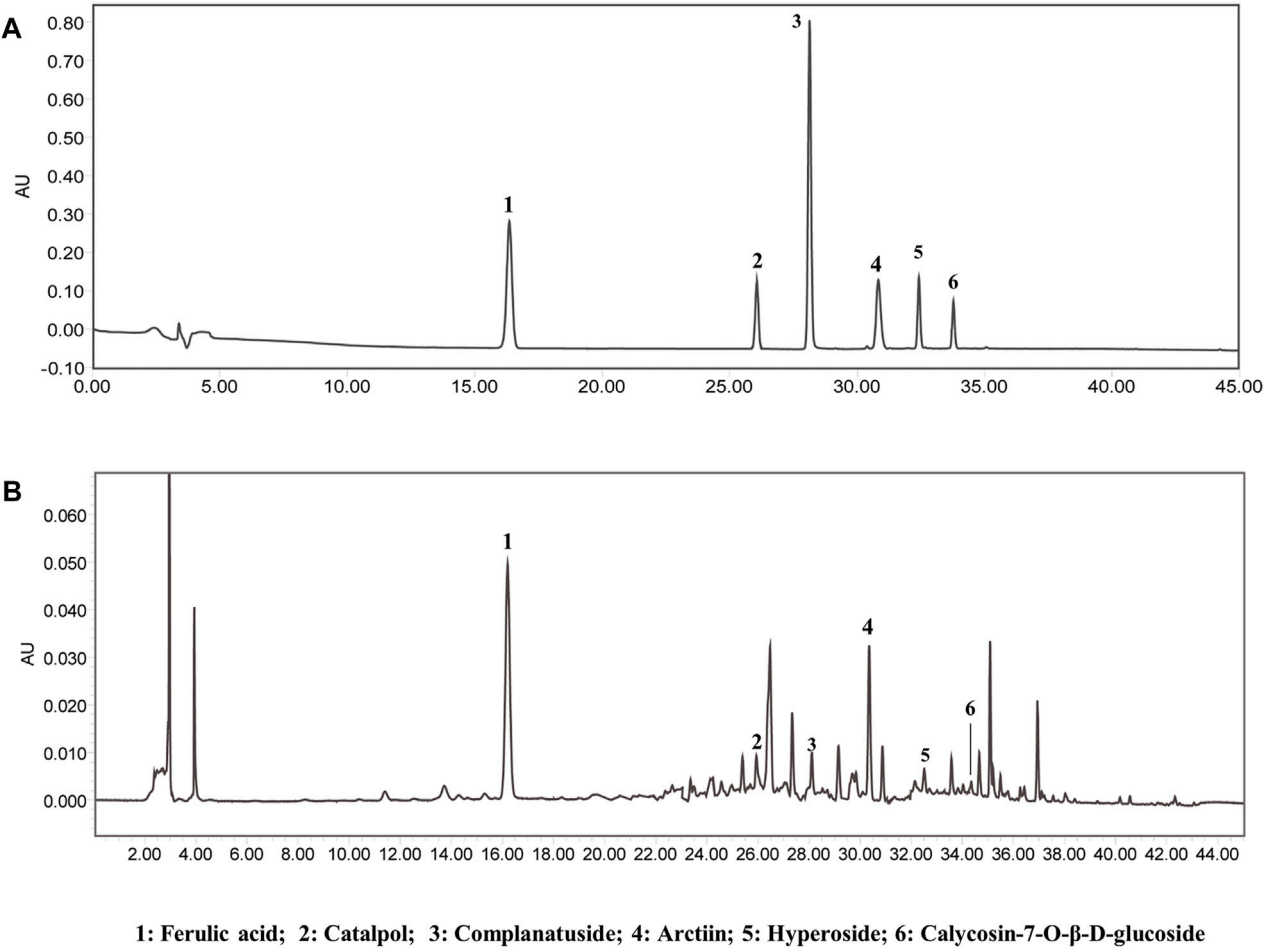
Chemical profiling of YC. The HPLC analysis showed that the major components of YC include ferulic acid, paeoniflorin, liquiritin, glycyrrhizic acid, and ligustilide. **(A)** Standards. **(B)** Samples.

The main components of YC are ferulic acid, paeoniflorin, liquiritin, glycyrrhizic acid, and ligustilide. As shown in Figures 1A,B, these six components were identified in YC by comparative analysis of the molecular retention times of the standards and YC samples, ferulic acid 2.175 mg/kg, catalpol 0.485 mg/kg, complanatuside 0.292 mg/kg, arctiin 6.074 mg/kg, hyperoside 0.101 mg/kg, and calycosin-7-O-β-D-glucoside. In addition, Supplementary Figure S1; Supplementary Table S1 showed that the LC-MS analysis in negative mode detected 10 sample peaks in the liquid chromatogram of the YC extract, which corresponded to ferulic acid, rehmannioside D, catalpol, complanatoside, epimedin A, epimedin B, arctiin, hyperoside, calycosin-7-O-beta-D-glucoside, astragaloside A.

### Effect of YC on ornidazole-induced oligoasthenozoospermia *in vivo*

**FIGURE 2:**
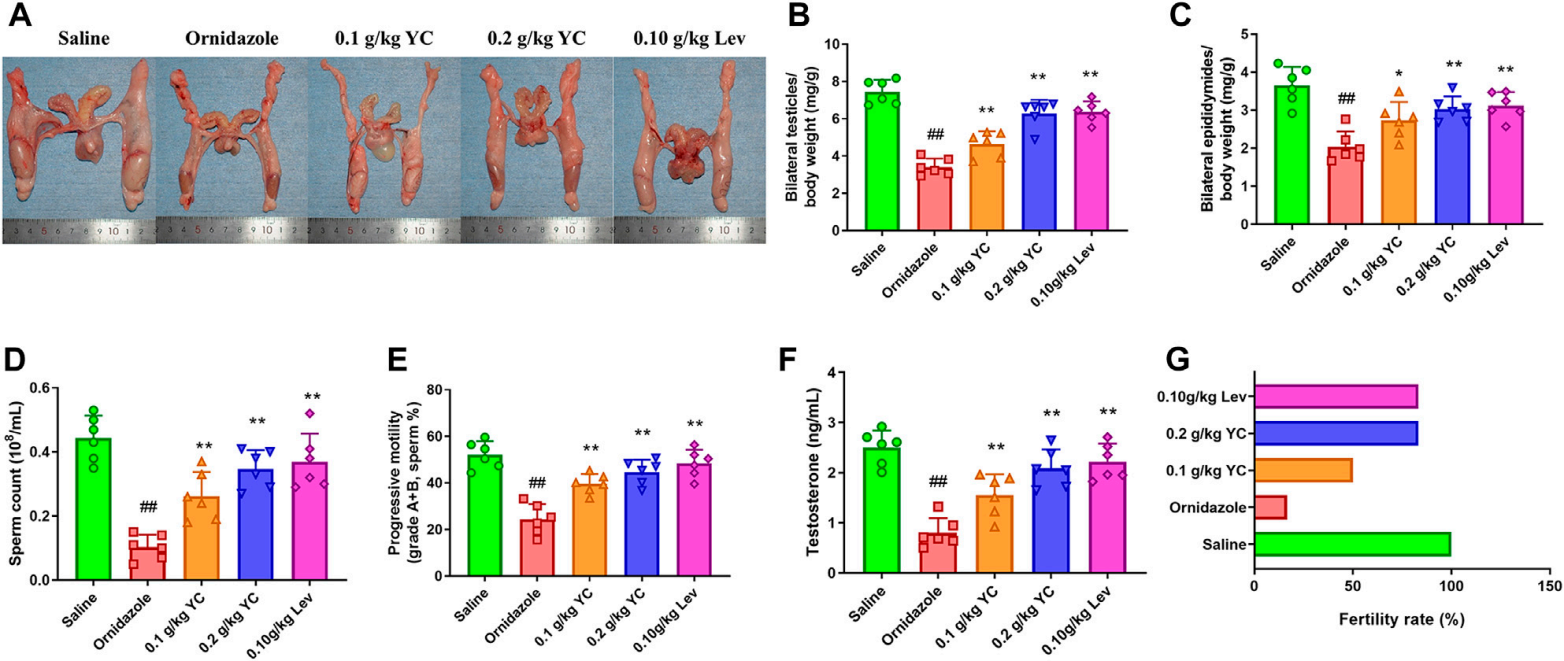
Effects of YC on oligoasthenozoospermia in rats exposed to ornidazole. **(A)** Characteristics of reproductive organs. **(B)** Relative weights of bilateral testicles. **(C)** Relative weights of bilateral epididymides. **(D, E)** Sperm concentration and progressive motility in the epididymis. **(F)** Levels of testosterone in the serum. **(G)** The fertility rate in percentages. Values are shown as averages ± SD (n = 6). ##*p* < 0.01 *versus* Saline group; *p < 0.05, **p < 0.01 *versus* Ornidazole group.

**FIGURE 3:**
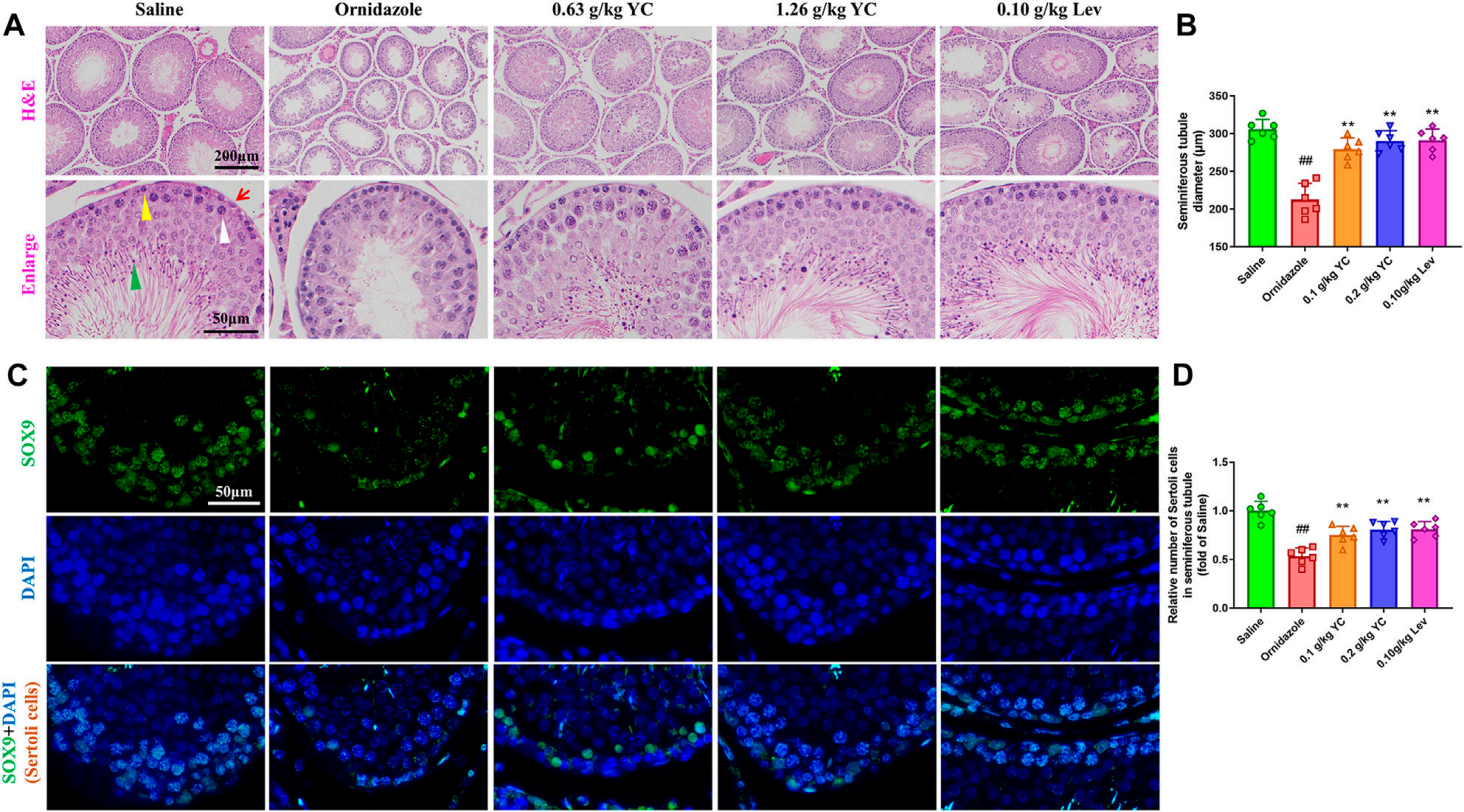
Testis features in the H&E staining and immunofluorescent analysis. **(A)** Representative images of hematoxylin-eosin-stained testis sections. Red arrows denote Sertoli cells; yellow triangles denote spermatogonia; white arrows denote primary spermatocytes; and green arrows denote spermatids. **(B)** Quantification of the diameters of seminiferous tubules in the H&E staining. **(C, D)** Representative images and quantification of SOX9-labelled Sertoli cells in the seminiferous tubule. Values are shown as averages ± SD (*n* = 6). ##*p* < 0.01 *versus* Saline group; **p < 0.01 *versus* Ornidazole group.

To determine the function of YC in oligoasthenozoospermia *in vivo*, the testes of rats were dissected for weight measurement and sperm analysis. As shown in Figures 2A–E, YC and levocarnitine greatly reversed ornidazole-induced reductions in bilateral testicles and epididymides weight, as well as the epididymal sperm concentration and progressive motility. Moreover, serum testosterone levels were restored after YC or levocarnitine treatment (Figure 2F). We also evaluated the fertility of male rats by conducting the fertility assay (Figure 2G). The results showed that ornidazole-treated rats displayed lower fertility rates as compared to the normal control group. Meanwhile, a higher fertility rate was observed in the YC- and levocarnitine-treated groups when compared with normal saline-treated model rats. However, the number of litter was not affected (data not shown). In the histological examination, spermatogonia, primary spermatocytes, and the diameter of the seminiferous tubule were decreased due to treatment with ornidazole, which was reversed by YC or levocarnitine intervention (Figures 3A,B). In addition, the immunofluorescent staining indicated that YC and levocarnitine inhibited ornidazole-induced loss of SOX9 (a key Sertoli cell protein)-labeled Sertoli cells in the seminiferous tubule (Figures 3C,D). Collectively, these findings suggested that similar to levocarnitine, YC improved ornidazole-induced oligoasthenozoospermia *in vivo.*

### YC boosts NO levels by activating the PLCγ1/AKT/eNOS pathway *in vivo*

**FIGURE 4:**
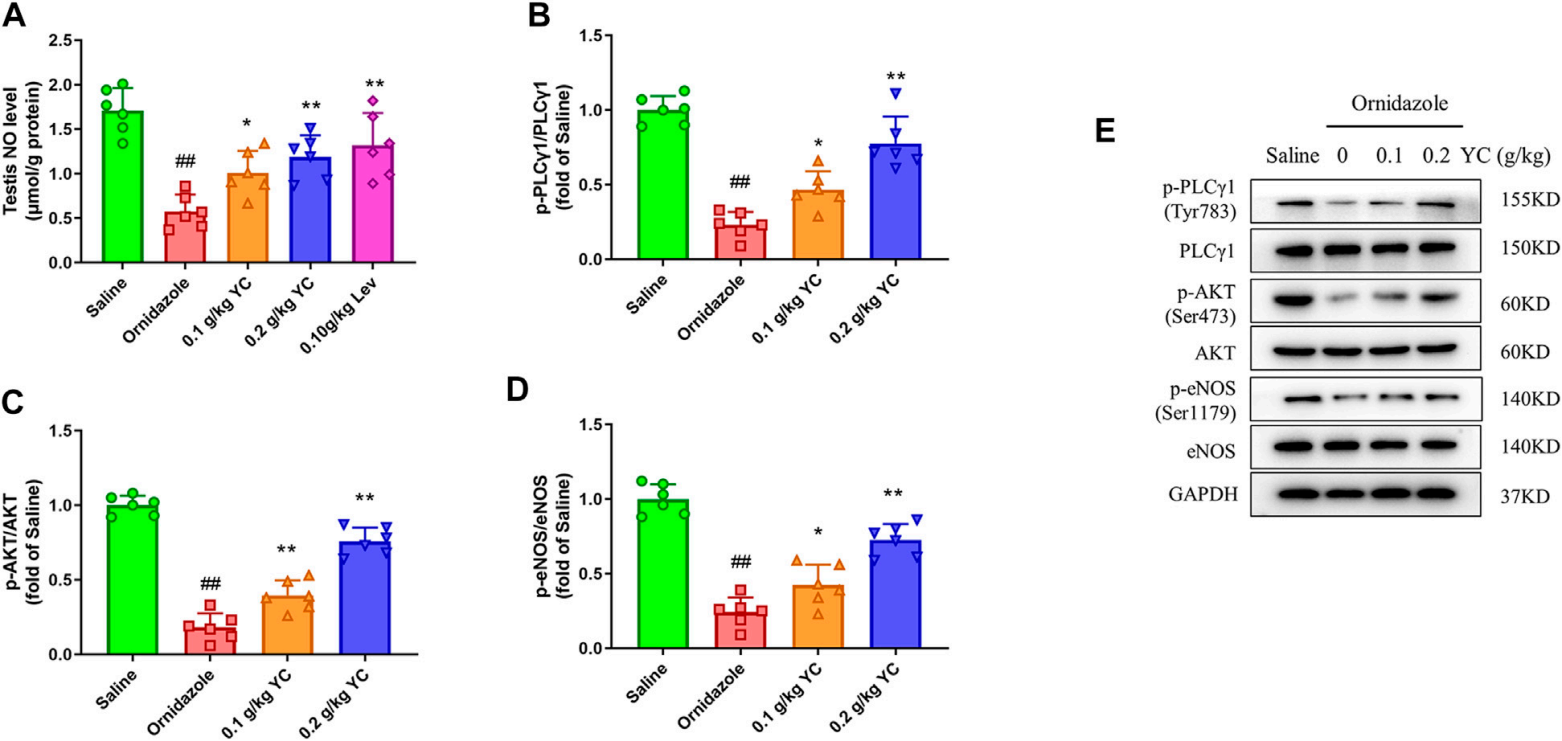
Nitric oxide (NO) concentrations and protein expression of PLCγ1/AKT/eNOS signaling in the testis. **(A)** NO levels in the testes were measured by using the corresponding commercial kit. **(B–E)** Protein expression of p-PLCγ1, p-AKT and p-eNOS in the testis. Values are shown as averages ± SD (*n* = 6). ##*p* < 0.01 *versus* Saline group; *p < 0.05, **p < 0.01 *versus* Ornidazole group.

Next, we explored whether YC attenuated oligoasthenozoospermia by boosting NO concentration through the PLCγ1/AKT/eNOS signaling pathway. As shown in Figures 4A–E, the decrease in testicular NO concentration induced by ornidazole was increased by YC. Furthermore, YC increased the phosphorylation of PLCγ1, AKT and eNOS in the testis. This demonstrated that the PLCγ1/AKT/eNOS signaling-mediated NO generation was involved in the anti-oligoasthenozoospermia effect of YC *in vivo.*

### YC inhibits ornidazole-induced oligoasthenozoospermia *in vitro*

**FIGURE 5:**
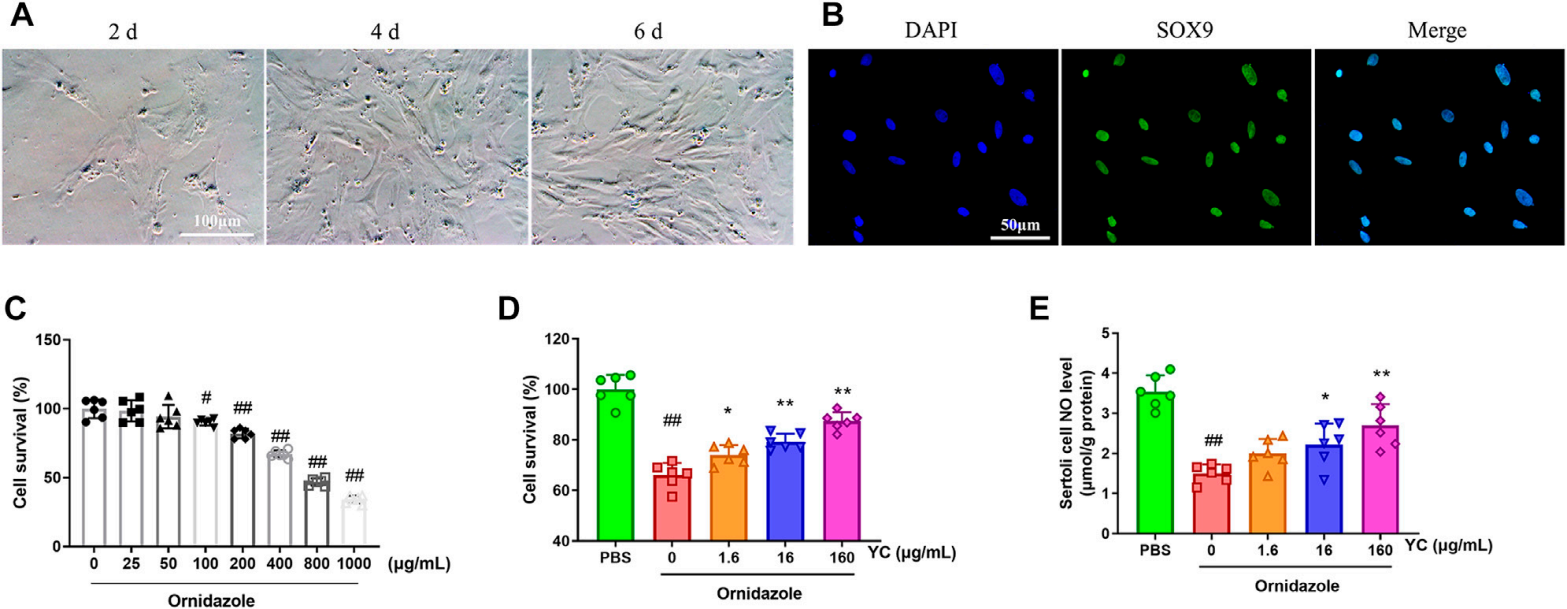
Effects of YC on ornidazole-induced oligoasthenozoospermia in primary Sertoli cells. **(A)** Cell morphology of primary Sertoli cells on different days after isolation. **(B)** Representative immunostaining staining of SOX9 positive (primary Sertoli cells) counterstained with DAPI. The purity of primary Sertoli cells was >98%. **(C)** Primary Sertoli cells were stimulated with ornidazole for 24 h, and viability was assessed by the MTT assay. **(D)** Primary Sertoli cells were treated with YC extract for 24 h, followed by 400 μg/mL ornidazole for another 24 h. Cell viability was assessed by the MTT assay. **(E)** NO levels in Sertoli cells were detected using the corresponding commercial kit. Values are shown as averages ± SD (*n* = 6). #*p* < 0.05, ##*p* < 0.01 *versus* the blank or PBS group; *p < 0.05, **p < 0.01 *versus* the Ornidazole + YC (0) group.

**FIGURE 6:**
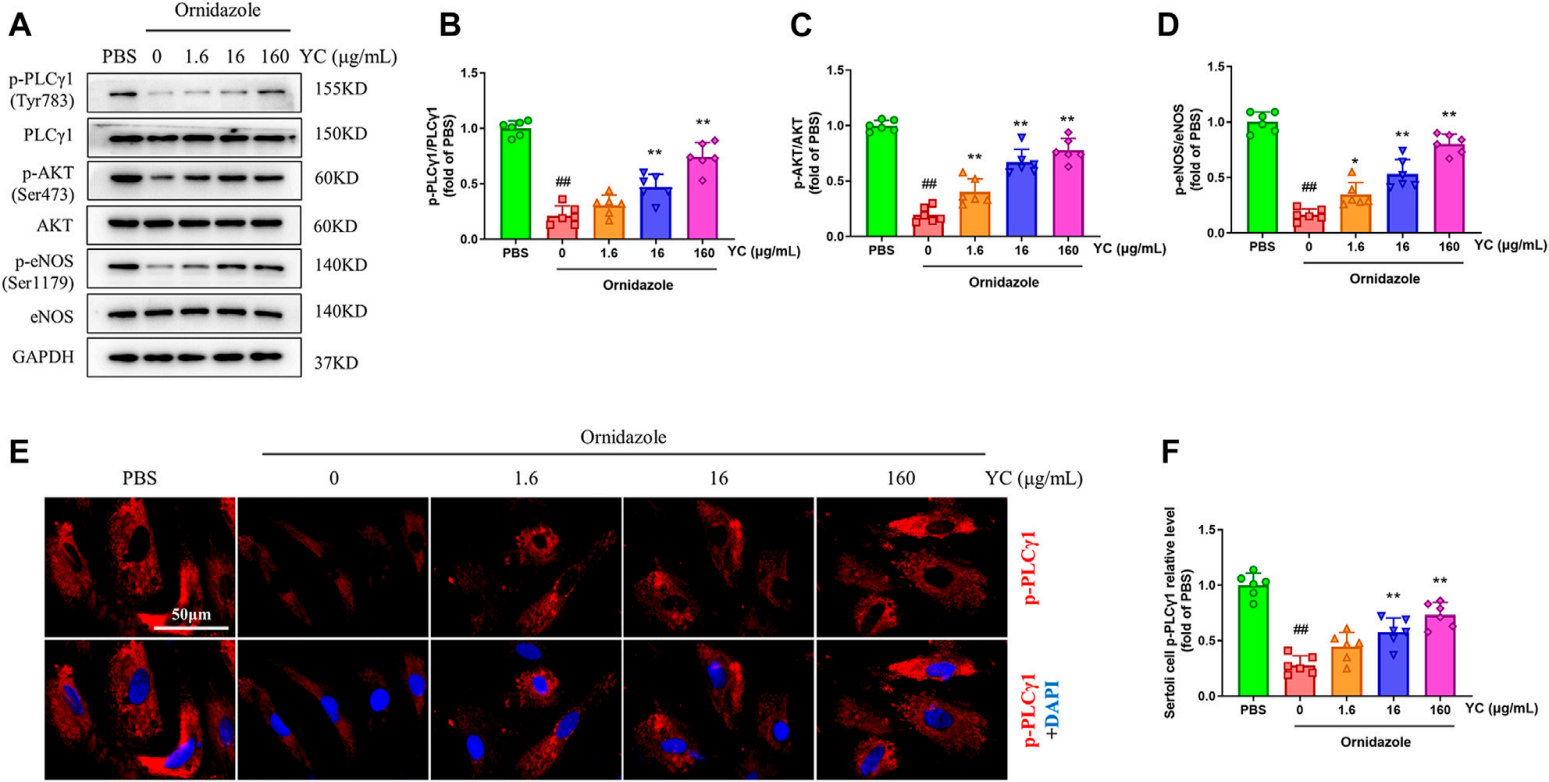
Changes of PLCγ1/AKT/eNOS signaling in ornidazole-stimulated primary Sertoli cells. **(A–D)** Protein levels of p-PLCγ1, p-AKT and p-eNOS were determined using western blot analysis. **(E, F)** Representative images and quantification of p-PLCγ1 levels in the immunofluorescence assay. Values are shown as averages ± SD (*n* = 6). ##*p* < 0.01 *versus* PBS group; *p < 0.05, **p < 0.01 *versus* Ornidazole + YC (0) group.

To assess the effect of YC *in vitro*, primary Sertoli cells were treated with YC extract for 24 h, followed by 400 μg/mL ornidazole (which decreased cell survival to 67%) for another 24 h (Figures 5A–C). The MTT assay results revealed that ornidazole greatly reduced the cell viability of Sertoli cells (Figure 5D). However, YC significantly reversed this decline, indicating the protective effect of YC on oligoasthenozoospermia *in vitro*. Furthermore, NO generation, the protein expression of p-PLCγ1, p-AKT and p-eNOS, and the relative intensity of p-PLCγ1 were reduced following ornidazole exposure, but were all enhanced after treatment with YC (Figure 5E; Figures 6A–F). This demonstrated that the PLCγ1/AKT/eNOS signaling-mediated NO production might be implicated in the anti-oligoasthenozoospermia effect of YC *in vitro.*

### PLCγ1 knockdown blocks YC-Induced restoration of the PLCγ1/AKT/eNOS pathway

**FIGURE 7:**
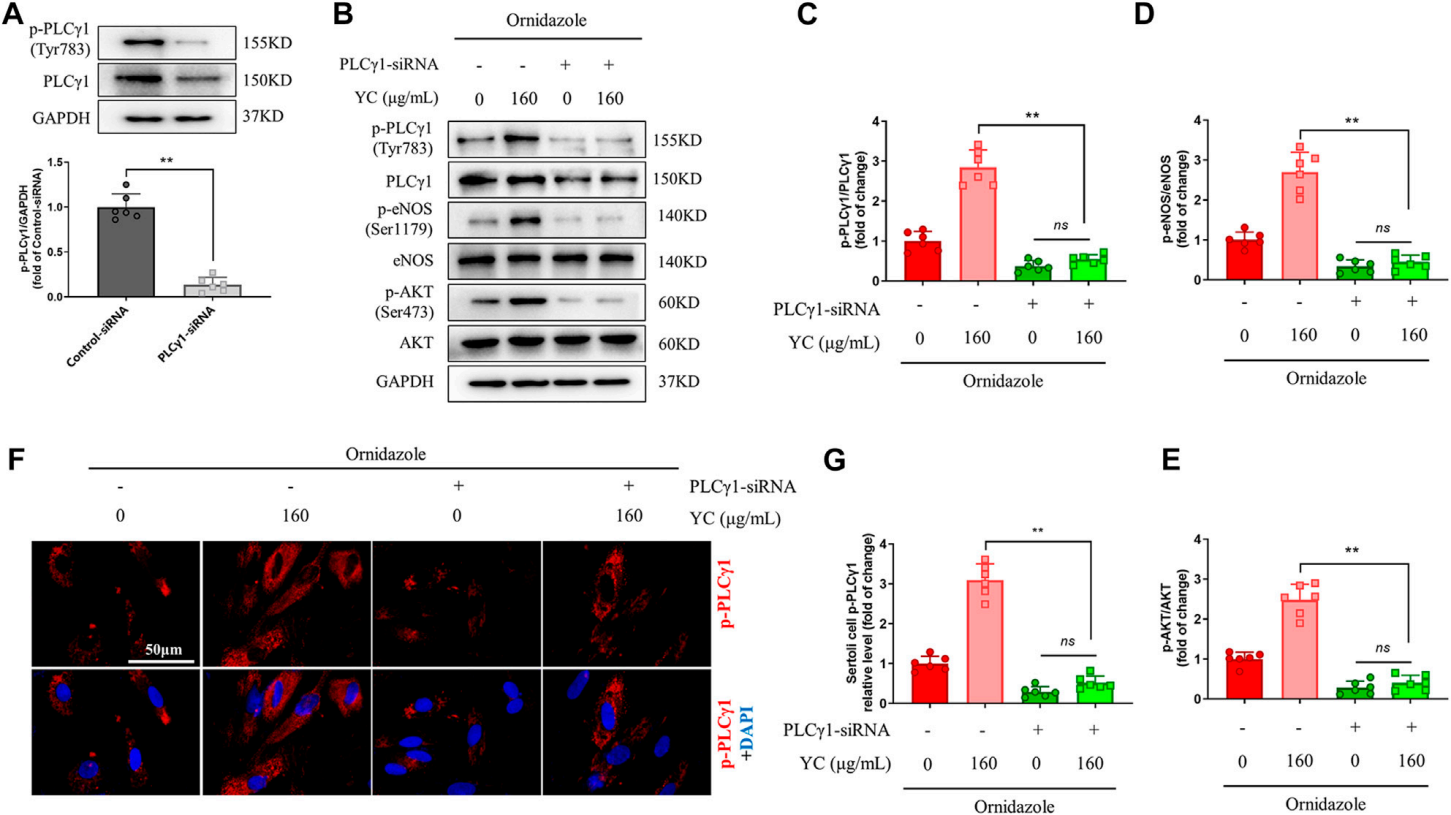
PLCγ1-siRNA compromises YC-induced improvement in primary Sertoli cells exposed to ornidazole. **(A)** PLCγ1-siRNA transfection efficiency was measured using the western blot assay. **(B–E)** Protein expression of p-PLCγ1, p-eNOS and p-AKT in the western blot analysis. **(F, G)** Representative images and quantification of p-PLCγ1 levels in the immunofluorescence assay. Values are shown as averages ± SD (*n* = 6). **p < 0.01; ns, not significant.

PLCγ1-siRNA was used to examine whether PLCγ1 mediated the effects of YC on Sertoli cells under an oligoasthenozoospermic condition. Compared with the control-siRNA group, Sertoli cells transfected with PLCγ1-siRNA had significantly lower p-PLCγ1 protein levels, indicating the success of PLCγ1-siRNA transfection (Figure 7A). Moreover, western blotting and immunofluorescent staining revealed that YC inhibited the downregulation of p-PLCγ1, p-eNOS, and p-AKT in ornidazole-stimulated Sertoli cells (Figures 7B–G). However, these effects were abolished by PLCγ1-siRNA transfection. These data indicated that PLCγ1 is necessary for YC-induced anti-oligoasthenozoospermia efficacy.

## Discussion

It is reported that approximately 50% of the 50 million infertile couples suffering from infertility worldwide have difficulty conceiving due to male factors (Lundy et al., 2021). Oligoasthenozoospermia is a major pathological contributor to this condition, where oligospermia and asthenozoospermia occur simultaneously (Bai et al., 2021). Unfortunately, effective pharmaceutical treatments for oligoasthenozoospermia are lacking in western medicine (Zhao et al., 2018). In recent years, attention has been drawn to the use of traditional Chinese medicine (TCM), which has been practiced for more than 2,000 years and gained widespread clinical applications (Hao et al., 2017). A meta-analysis of randomized controlled trials revealed that Wuzi Yanzong pill, a TCM formula, decreases DNA damage and improves sperm concentration, sperm motility, and the activity of the acrosomal enzyme in patients with oligoasthenozoospermia (Zhao et al., 2018). Huatan Qushi decoction rescues oligoasthenozoospermia through the improvement of lipid metabolism and semen quality in obese rats (Dong et al., 2019). Astragalin, one of the main active ingredients in TCM, alleviates oligoasthenozoospermia by increasing testosterone levels in the testis (Fan et al., 2022). Previously, Gu et al. reported the stimulative effect of YC on testosterone synthesis via the Nur77 pathway in Leydig cells (Gu et al., 2015). Zhao et al. found that YC ameliorates spermatogenesis in male mice exposed to cyclophosphamide (Zhao et al., 2015). In the current study, YC increased the sperm parameters, serum testosterone concentration, and fertility rate in rats exposed to ornidazole, demonstrating the anti-oligoasthenozoospermia property of YC and further supporting the potential of YC in treating male infertility.

Nitric oxide (NO), an unorthodox messenger molecule synthesized by nitric oxide synthase (NOS), plays a critical role in infertility. Najafi et al. measured the protein and mRNA expression of eNOS in women with unexplained infertility and observed the alteration of eNOS in the endometrium (Najafi et al., 2012). NO affects vascular changes and tissue remodeling during ovulation and luteinization; ovulation in rats could be decreased by NOS inhibitors, such as AG and L-NAME (Luo et al., 2021). The work conducted by Pozzi and his colleagues showed that NO donor drugs might play a beneficial role in restoring the balance between ROS synthesis and degradation, contributing to the improvement of overall fertilization capacity (Pozzi et al., 2021). In addition, the NOS/NO pathway was proven to regulate the junction integrity in the seminiferous epithelium, control the levels of hormones and cytokines in the testis, and modulate germ cell viability and development (Lee and Cheng, 2008). A case-control study revealed that NOS3 rs1799983, a NOS gene polymorphism, increases the risk of oxidative sperm DNA damage, which leads to male infertility (Yan et al., 2014). NO is also a unique mediator in oligoasthenozoospermia. The endothelial nitric oxide synthase (Glu298Asp variant) affects seminal parameters in men with idiopathic oligoasthenozoospermia (Delli Muti et al., 2014). When compared to the normospermic group, the NO concentration in the seminal plasma of oligoastenoteratospermic men is considerably lower (Kalezic et al., 2018). In individuals diagnosed with asthenozoospermia, NO levels and the proportion of immotile spermatozoa have been reported to be positively correlated (Buldreghini et al., 2014). Ginsenoside R(e) markedly increased asthenozoospermic infertile human sperm motility by boosting NO production and NOS activity; however, the NOS inhibitor N (omega)-Nitro-L-arginine methyl ester (L-NAME) or NO scavenger N-Acetyl-L-cysteine (LNAC) inhibited the actions of Ginsenoside R (e) (Zhang et al., 2006). Here, we found that YC improved the fertility rate of ornidazole-exposed rats through the improvement of sperm concentration and progressive motility via the NO level, confirming the essential role of NO in ameliorating oligoasthenozoospermia.

The PLCγ1/AKT/eNOS signaling is an important pathway implicated in NO production and oligoasthenozoospermia. Erythropoietin-induced phosphorylation of PLCγ1 contributes to nitric oxide (NO) production, phosphorylation of eNOS and AKT, and formation of the TRPV1-AKT-AMPK-eNOS complex; however, the specific antagonist of PLCγ1 compromised the erythropoietin-induced eNOS phosphorylation, TRPV1-eNOS complex formation and NO production (Yu et al., 2017). Previous research revealed that PLCγ1 activity affects the shaping of spermatozoa. (Schnabel et al., 2005). Using the network pharmacology technique, Chen et al. discovered that the AKT pathway has a substantial role in oligoasthenozoospermia and was associated with the Yishen Tongluo formula used to treat the condition (Dong et al., 2022). Panax ginseng glycoproteins regulate AKT signaling and promote sex hormones, sperm quality, and the differentiation process in a murine model of oligoasthenozoospermia (Shan et al., 2021). The study performed by Song and his colleagues suggested that the eNOS gene variants T-786C and 4a4b loci are risk factors for idiopathic asthenozoospermia in both Asian and Caucasian populations (Song et al., 2015). By regulating the eNOS pathway, histamine and quercetagetin reversed deltamethrin-induced germ cell apoptosis and reduced sperm production in male SD rats (Yu et al., 2014). Moreover, enhanced testicular eNOS expression induced by tangeretin was shown to improve epididymal sperm concentration and motility as well as seminiferous tubule morphology in hypertensive rats (Chiangsaen et al., 2020). In our work, YC mitigated ornidazole-induced oligoasthenozoospermia through the PLCγ1/AKT/eNOS signaling, while the blockage of PLCγ1 blunted its beneficial effects in ornidazole-induced Sertoli cells, providing further evidence for the involvement of the PLCγ1/AKT/eNOS pathway in oligoasthenozoospermia. The study has certain limitations. First, we only explored the protective effect of YC in chemical injury (ornidazole)-induced oligoasthenozoospermia. However, other factors such as infections, genetic abnormalities, endocrine disorders, and testicular dysfunction can also cause oligoasthenozoospermia. Thus, more models are needed to fully evaluate YC-generated beneficial effects in oligoasthenozoospermia. Second, in this work, we found that YC mitigated ornidazole-induced oligoasthenozoospermia via PLCγ1/AKT/eNOS signaling. Previously, YC has been demonstrated to enhance the function of testicular angiogenesis by activating the VEGFA/eNOS pathway (Jin et al., 2020). In addition, AKT has been demonstrated to regulate VEGFA expression (Peng et al., 2021). Further experiments are needed to find out if YC improves oligoasthenozoospermia by regulating the VEGFA/eNOS pathway via the PLCγ1/AKT axis.

## Conclusion

The present study suggested that YC conferred protective effects on oligoasthenozoospermia by boosting NO levels through the PLCγ1/AKT/eNOS pathway. Moreover, PLCγ1 signaling might be a potential target for the treatment of oligoasthenozoospermia.

## Data Availability

The original contributions presented in the study are included in the article/Supplementary Material, further inquiries can be directed to the corresponding author.

## Abbreviations

ANOVA, analysis of variance; YC, Yangjing capsule; SD, Sprague-Dawley; PLCγ1, Phospholipase C γ1; NO, nitric oxide; NOS, nitric oxide synthase; nNOS, neuronal nitric oxide synthase; eNOS, endothelial nitric oxide synthase; iNOS, inducible nitric oxide synthase; EDTA, ethylene diamine tetra-acetic acid; FBS, fetal bovine serumDMEM, Dulbecco’s modified Eagle medium; H&E, hematoxylin and eosin; BSA, bovine serum albumin; BCA, bicinchoninic acid; PVDF, polyvinylidene fluoride; MTT, methyl thiazolyl tetrazolium; DMSO, dimethyl sulfoxide; SD, standard deviation.
