# Supplementary material for: Yangjing capsule improves oligoasthenozoospermia by promoting nitric oxide production through PLCγ1/AKT/eNOS pathway
Source: Front Pharmacol. 2023 Apr 26;14:1056091. doi: 10.3389/fphar.2023.1056091 (PMC10169610; doi:10.3389/fphar.2023.1056091)
Supplement: Supplementary file 1 [file DataSheet1.docx]

Figure S1 Representative base peak intensity chromatograms of standard compounds (A) and Yangjing capsule (B) analyzed by LC-MS/MS in negative mode.


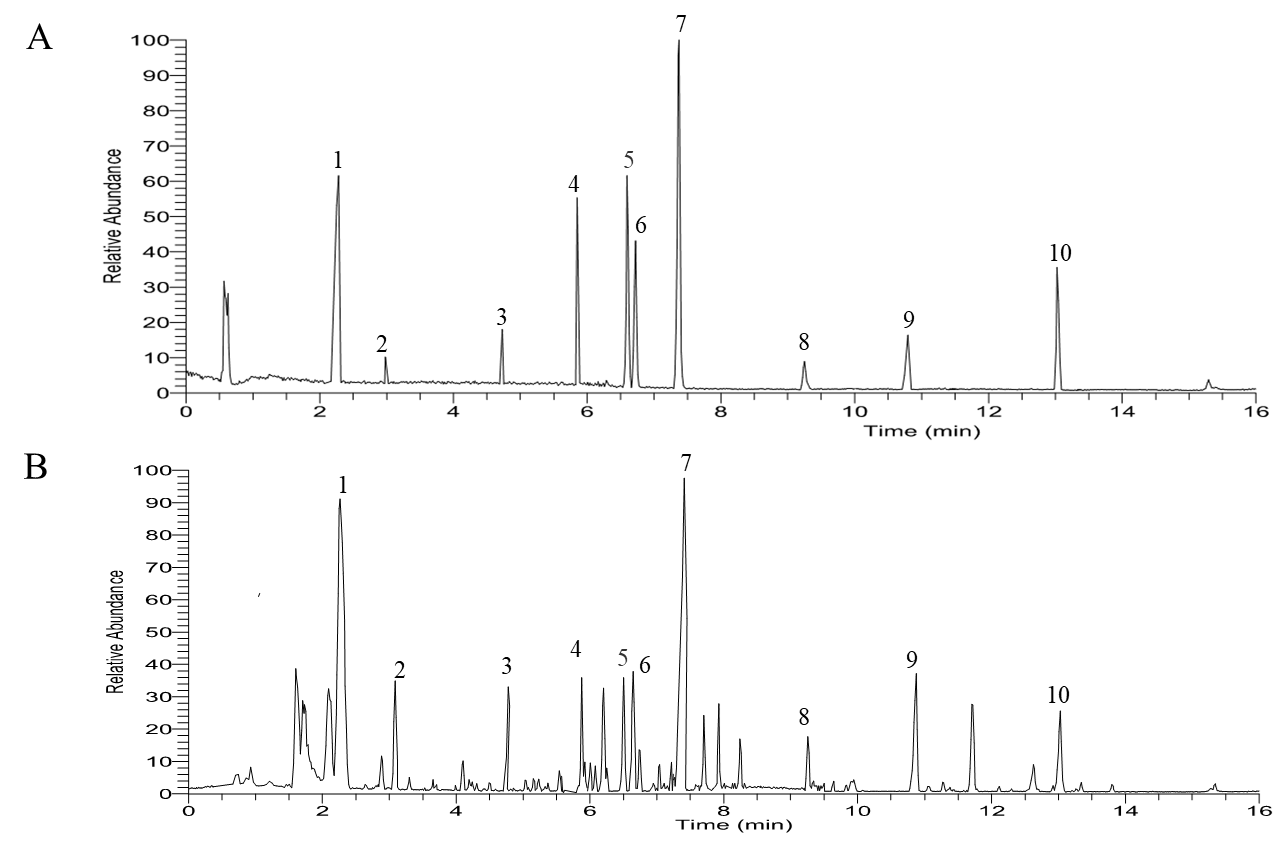


Table S1 Identified compounds and their corresponding parameters by LC-MS/MS in negative mode

| **Number** | **Name** | **Formula** | **Mass (m/ z)** |
| --- | --- | --- | --- |
| 1 | Ferulic acid | C10H10O4 | 193 |
| 2 | Rehmannioside D | C27H42O20 | 685 |
| 3 | Catalpol | C15H22O10 | 361 |
| 4 | Complanatoside | C27H32O14 | 623 |
| 5 | Epimedin A | C39H50O20 | 838 |
| 6 | Epimedin B | C38H48O19 | 808 |
| 7 | Arctiin | C27H34O11 | 533 |
| 8 | Hyperoside | C21H20O12 | 463 |
| 9 | Calycosin-7-O-beta-D-glucoside | C22H22O10 | 445 |
| 10 | Astragaloside A | C41H68O14 | 784 |
